# Sex and Heart Failure with Preserved Ejection Fraction: From Pathophysiology to Clinical Studies

**DOI:** 10.3390/jcm8060792

**Published:** 2019-06-04

**Authors:** Marijana Tadic, Cesare Cuspidi, Sven Plein, Evgeny Belyavskiy, Frank Heinzel, Maurizio Galderisi

**Affiliations:** 1 Department of Internal Medicine and Cardiology, Charité–Universitätsmedizin Berlin, Augustenburgerplatz 1, 13353 Berlin, Germany; Evgeny.Belyavskiy@charite.de (E.B.); Frank.heinzel@charite.de (F.H.); 2 Clinical Research Unit, University of Milan-Bicocca and Istituto Auxologico Italiano, Viale della Resistenza 23, 20036 Meda, Italy; cesare.cuspidi@unimib.com; 3 Department of Biomedical Imaging Science, Leeds Institute of Cardiovascular and Metabolic Medicine, University of Leeds, Leeds LS2 9JT, UK; s.plein@leeds.ac.uk; 4 Department of Advanced Biomedical Sciences, Federico II University Hospital, Edificio 1 Via Pansini 5, 80131 Naples, Italy; Maurizio.gaderi@unina.com

**Keywords:** HFpEF, sex, epidemiology, risk factors, treatment

## Abstract

Heart failure with preserved ejection fraction (HFpEF) represents the most frequent form of heart failure in women, with almost two-fold higher prevalence than in men. Studies have revealed sex-specific HFpEF pathophysiology, and suggested the possibility of a sex-specific therapeutic approach in these patients. Some cardiovascular risk factors, such as arterial hypertension, obesity, diabetes mellitus, coronary artery disease, atrial fibrillation, and race, show specific features that might be responsible for the development of HFpEF in women. These risk factors are related to specific cardiovascular changes—left ventricular diastolic dysfunction and hypertrophy, ventricular–vascular coupling, and impaired functional capacity—that are related to specific cardiac phenotype and HFpEF development. However, there is no agreement regarding outcomes in women with HFpEF. For HFpEF, most studies have found higher hospitalization rates for women than for men. Mortality rates are usually not different. Pharmacological treatment in HFpEF is challenging, along with many unresolved issues and questions raised. Available data on medical therapy in patients with HFpEF show no difference in outcomes between the sexes. Further investigations are necessary to better understand the pathophysiology and mechanisms of HFpEF, as well as to improve and eventually develop sex-specific therapy for HFpEF.

## 1. Introduction

Heart failure (HF) represents a large health burden because of its increasing prevalence, significant morbidity and mortality, and constantly increasing expenses of treatment [1]. The introduction of new entities—heart failure with preserved ejection fraction (HFpEF), and, later, heart failure with mid-range ejection fraction (HFmEF)—has significantly broadened the HF spectrum. Studies published in the last few years have revealed that cardiovascular disease (CVD) is the main cause of death among women, and more women than men die from CVD [2]. For HFpEF, there is clear evidence of important differences between the sexes. Namely, the prevalence of HFpEF ranges between 40 and 70% of all patients with HF, but the ratio between women and men is 2:1 [3,4]. Several authors have tried to ascribe this difference to comorbidities such as obesity and diabetes, which are more prevalent in women. However, this is not sufficient to explain the significantly higher HFpEF prevalence and mortality in women. The main problem for the assessment of sex influence on HFpEF morbidity and mortality is the fact that, in majority of published studies, the assessment of the sex effect was retrospective.

The data regarding differences in pathophysiology, outcomes, and treatment in women and men with HFpEF are scarce. One of the main problems in the assessment of sex and its impact on HFpEF morbidity and mortality is the retrospective design of most studies.

PubMed, Medline, OVID, Cochrane, and EMBASE databases were searched for studies published in the English language from January 1990 through to February 2019, using the following keywords: “heart failure with preserved ejection fraction,” “HFpEF,” “gender,” “sex,” “prevalence,” “risk factors,” “left ventricle,” “diastolic function,” “renin-angiotensin inhibitors,” “angiotensin receptor blockers,” “beta-blockers,” and “aldosterone.”

The aim of this systematic review article is to provide a comprehensive overview of currently known disparities in epidemiology, pathophysiology, and recent clinical studies regarding hemodynamic changes, cardiac remodeling, treatment, and outcome.

## 2. Epidemiology

The latest report of the American Heart Association included 110,621 HF patients, 50% (55,083) of whom had HF with reduced EF (HFrEF), 14% (15,184) HFmEF, and 36% (40,354) HFpEF. The report emphasized three important findings: HFpEF was more prevalent in women compared with men, there was no difference in HFpEF incidence between the sexes, and HFpEF was the most common subtype in women with HF [5]. These data agree with other recently published studies. Goyal et al. included 1,889,608 hospitalizations due to HFpEF, and showed that the prevalence of hospitalizations was significantly higher among women than men (64% vs. 36%) [6]. Harada et al. also reported significantly higher prevalence of HFpEF in women than in men (529 women vs. 204 men, *p* < 0.001) [7]. Duca et al. confirmed these findings in a small study that included 260 HFpEF patients (181 females and 79 males) [8]. It should be emphasized that the majority of trials that investigated the effect of medications on HFpEF performed adjustments for sex in order to account for sex-related effects [9].

A recently published study revealed that the lifetime risk of HFpEF at index ages 45 through 90 was similar in men and women, whereas the lifetime risk of HFrEF was 1.8-fold higher in men compared with women [10]. The lifetime risks of HFpEF and HFrEF were similar in men while women had a substantially higher lifetime risk of HFpEF than HFrEF [10]. Interestingly, the lifetime risks of HFpEF and HFrEF were not substantially lower at higher index ages in men and women. Overall, current evidence clearly shows that sex-specific prevalence exists in HFpEF patients.

## 3. Pathophysiology

**Figure 1:**
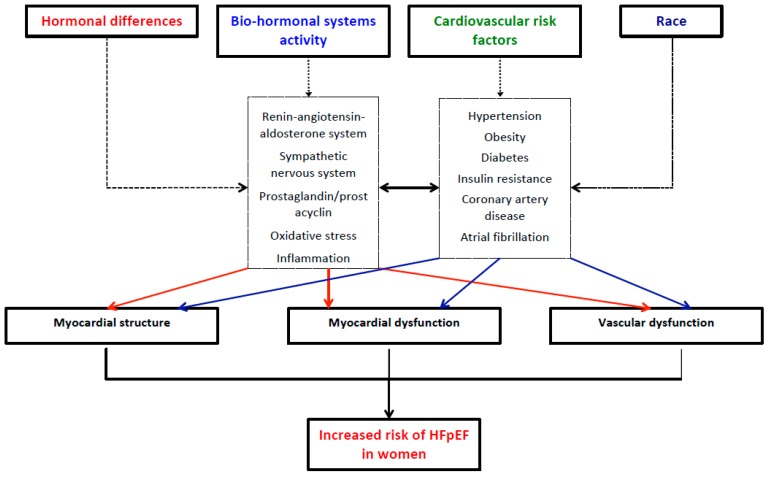
Sex differences in HFpEF development.

**Figure 2:**
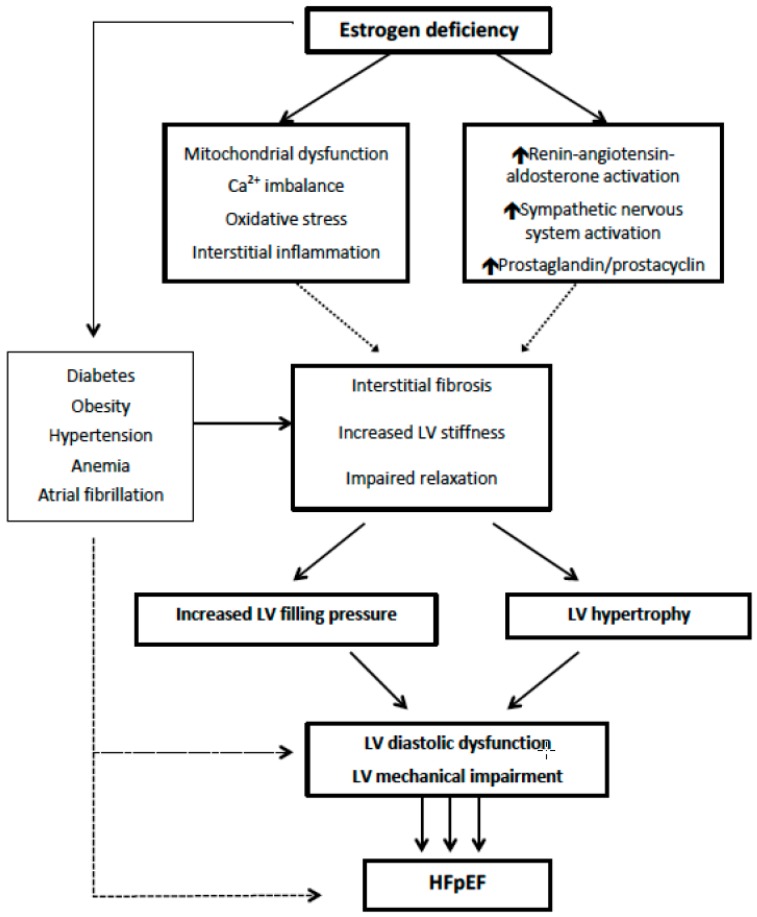
The effect of estrogen deficiency in the pathophysiology of HFpEF.

Pathophysiological mechanisms that could explain sex-related differences in HFpEF can be separated into several groups: (i) hormonal differences; (ii) bio-hormonal system activity (renin-angiotensin-aldosterone system, sympathetic nervous system, prostaglandin/prostacyclin, oxidative stress, inflammation); (iii) differences in cardiovascular risk factors and predisposing diseases important for HFpEF development (hypertension, obesity, diabetes and insulin resistance, coronary artery disease, atrial fibrillation); and (iv) race, which has been an important predictor of HFpEF development or worse outcome in HFpEF patients in recent studies. The central illustration (Figure 1) illustrates the proposed sex-specific pathophysiological mechanisms responsible for HFpEF development in women. Figure 2 shows the influence of estrogen deficiency on left ventricular (LV) remodeling and development of HFpEF. All these mechanisms could induce a sex-specific hemodynamic response, and, ultimately, different functional and structural LV remodeling in men and women, which could partly explain the variations in HFpEF induced by sex.

### 3.1. Hormonal Differences

The MESA (Multi-ethnic study of Atherosclerosis) study, after a nine year follow-up, reported that the androgenic profile, characterized by higher free testosterone and lower sex hormone binding globulin, was related to a higher increase in LV mass in both sexes, whereas a greater increase in ratio between LV mass and volume was found only in women [11]. LVEF (Left ventricular ejection fraction) remained higher in women than in men for the whole duration of the study. These changes are consistent with the known effects of menopause, after which LV mass, but not LV volume, increases, leading to small and rigid LVs. Increased LV stiffness induces LV diastolic dysfunction and further HFpEF.

The same study showed that a higher testosterone/estradiol ratio and lower estradiol levels were associated with the increased risk of HFrEF, but not with HFpEF [12]. The authors hypothesized that the reduced estradiol during menopause affects vascular and cardiac remodeling, inducing more HFrEF than HFpEF. However, there are also many possible confounders in the MESA study that could interfere with the relationship between hormones and HFpEF occurrence.

Li et al. summarized the effects of estrogen on LV diastolic function: regulative function of mitochondria, cardiac hypertrophy, Ca^2+^ homeostasis, and titin isoform switches [13]. Considering the fact that LV diastolic dysfunction represents the cornerstone of HFpEF, the lack of estrogen could partly explain higher HFpEF in women.

### 3.2. Bio-Hormonal Systems

Bio-hormonal systems could be the leading causes of sex-specific differences in cardiovascular diseases. Studies have shown that sex differences exist in the aging pattern of the renin-angiotensin-aldosterone system (RAAS) [13]. Significantly lower angiotensin-converting enzyme (ACE) serum activity was found in older men compared to older women [14]. Furthermore, significantly lower ACE serum activity was detected in older men compared to younger men. In contrast, in women, there were no differences in ACE serum depending on age, but there was significantly higher ACE-2 serum activity in older women compared to younger women [14]. Thoering et al. demonstrated that men had a higher aldosterone level and a lower adrenal response to exogenous angiotensin II infusion than women [15]. These constitutive sex differences in the regulation of aldosterone were related to higher extracellular volume and higher blood pressure in men than in women [15]. 

RAAS is associated with LV hypertrophy as well as with LV diastolic dysfunction [16,17], and both are associated with LV remodeling, which finally induces HFpEF. These observations suggest mechanisms by which variations in bio-hormonal systems could induce sex variations in HFpEF patients, and may require sex-determined therapeutic approaches.

The sympathetic nervous system (SNS) is also sex-dependent in several ways: (i) the regulatory system for SNS activation is less sensitive to excitation and more sensitive to inhibition in women than in men; (ii) cardiopulmonary induced SNS inhibition is higher in women, which could result in better renal excretory function; (iii) reduced sensitivity to adrenergic nerve stimulation, but not to noradrenaline, indicates that sex-induced SNS variations could protect females against SNS hyperactivation; and (iv) women are less sensitive and/or less responsive to adrenal medullary activation, which helps them in the situations of increased stress [17,18,19].

Previous studies showed that SNS overactivity was related to impaired LV diastolic function [20] and LV hypertrophy [21], the major determinants of HFpEF. However, one should also not forget that RAAS and SNS overactivation are related to arterial hypertension, obesity, and diabetes/insulin resistance, which are main risk factors of HFpEF development in women [22,23,24].

Besides well-known bio-hormonal systems, systemic microvascular endothelial inflammation has lately been cited as an important risk factor of HFpEF [25]. Inflammation decreases nitric oxide bioavailability, the cyclic guanosine monophosphate level, and protein kinase G activity in cardiomyocytes. These changes induce interstitial fibrosis, and subsequently LV hypertrophy and elevated resting tone in cardiomyocytes due to hypophosphorylation of titin. Increased LV stiffness induces elevation of LV filling pressure and HFpEF development [25]. Recent studies have shown that sex-biased microRNA could be responsible for a different response to systemic inflammation that induces microvascular defects related to HFpEF [26]. Sex-biased microRNAs are regulated by estrogen in their transcription and processing, or are expressed from loci on the X chromosome due to incomplete X chromosome inactivation. Estrogen-induced microRNAs principally have a protective function, which is why menopausal estrogen deficiency results might contribute to the molecular mechanisms that increase the risk of HFpEF in women [26].

### 3.3. Cardiovascular Risk Factors

Obesity, hypertension, diabetes, coronary heart disease, atrial fibrillation, anemia, chronic obstructive pulmonary disease, renal dysfunction, and history of chemo- and radiotherapy are important risk factors of HFpEF development in both sexes, but it seems that some of these factors are more significant among females (Table 1). A recent study of 1204 HFpEF patients from Asia showed that 70% of the patients had ≥2 co-morbidities, most commonly hypertension (71%), anemia (57%), chronic kidney disease (50%), diabetes (45%), coronary artery disease (29%), atrial fibrillation (29%), and obesity (26%) [27]. In the next segment, we will particularly consider these comorbidities in the light of sex differences. 

#### 3.3.1. Hypertension

Hypertension represents one of the major factors responsible for HFpEF development [22]. Pandey et al. reported that hypertension was more prevalent in women than in men in all age groups [10]. However, it was statistically significantly higher only in the oldest group of patients (>75 years) [10]. Hoyal et al. reported that arterial hypertension was more prevalent in women than in men with HFpEF, and the difference was more pronounced in women older than 75 years of age [6]. Levi et al. reported similar findings, and calculated that hypertension increased the risk of HF three-fold in women, compared with two-fold in men [28]. However, at that time the HFpEF entity was unknown, and, therefore, the results relate to HFrEF. A large population of postmenopausal women showed that hypertension represented a significant risk factor of HFpEF development in women of all races (white, African American, and Hispanic) [29].

The importance of arterial hypertension in elderly women underlines the significance of isolated systemic hypertension, typical of this population of patients. A higher augmentation index between peripheral and central blood pressure in elderly women, compared with men, could explain more pronounced target organ damage, including LV hypertrophy [30]. Women have different adaptation to pressure overload than men, with more prevalent concentric than eccentric LV remodeling [31]. Higashi et al. reported a significant correlation between carotid augmentation index—as a parameter of arterial stiffness—and LV diastolic dysfunction only in women, but not men [32]. Mengden et al. summarized the relationship between isolated systolic hypertension, ventriculo-arterial coupling, and LV diastolic dysfunction in elderly women, and stated that this association could be the cornerstone of HFpEF in females [33]. 

#### 3.3.2. Obesity

Our study group has recently reviewed mechanisms that connect obesity and HFpEF, and we have emphasized the importance of sex in HFpEF development [23]. A large study followed 22,681 participants for 12 years, and revealed that body mass index (BMI) and insulin resistance were more strongly associated with the risk of HFpEF than HFrEF development in women, but not in men [34]. The same study showed that waist circumference was independently associated with HFpEF occurrence in both sexes. Goyal et al. demonstrated that obesity was significantly more prevalent among women than men with HFpEF [6]. However, Harada et al. showed that obesity, defined as a BMI > 25 kg/m^2^, was more prevalent in men than in women with HFpEF [7]. However, BMI correlated with plasma brain natriuretic peptide (BNP) only in women, but not in men with HFpEF [7].

In a large population of postmenopausal women whose obesity was followed for 13 years, there was an independent predictor of HFpEF, but not HFrEF [29]. A recently published study that investigated the impact of the pattern of regional adipose deposition (abdominal, cardiac, intermuscular) on cardiorespiratory fitness in patients with HFpEF revealed that abdominal subcutaneous fat was inversely associated with functional capacity in older patients with HFpEF [35]. Furthermore, intra-abdominal fat was the strongest independent predictor of reduced functional capacity in these patients [35]. This could explain prominent symptoms and increased mortality in obese women with HFpEF.

#### 3.3.3. Diabetes and Insulin Resistance

McHugh et al. have recently explained the complex relationship between diabetes and HFpEF [24]. The authors provided several mechanisms that included sodium retention and consequent volume overload, increased pro-inflammatory cytokines, poor skeletal muscle function, and impaired cardiorespiratory fitness [24].

Savji et al. reported that fasting glucose was an independent predictor of HFpEF occurrence only in women, but not in men [34]. Interestingly, the homeostatic model assessment (HOMA) index—a parameter of insulin resistance—was a predictor of HFpEF development only in men, but not in women [34]. Goyal et al. reported higher prevalence of diabetes only among younger patients (<75 years) hospitalized due to HFpEF [6]. Interestingly, in smaller studies that included fewer than 1000 HFpEF patients, the authors found significantly higher prevalence of diabetes among men than women [7,36], or reported no difference between the sexes [8].

#### 3.3.4. Coronary Artery Disease

Coronary artery disease is a dominant risk factor of the development of HFpEF in men. However, its influence in women should not be neglected. This was confirmed in large studies which showed that coronary artery disease, prior percutaneous coronary intervention, or coronary artery bypass grafting were significantly more frequent in men than in women hospitalized with symptoms of HFpEF [6]. Nakada et al. also reported that ischaemic heart disease was a more frequent cause of HFpEF among males than females (17.5% vs. 30.6%) [36]. Other authors reported similar findings by defining coronary artery disease as myocardial infarction [7,37]. Eaton et al. did not find a correlation between previous myocardial infarction and hospitalization due to HFpEF, but only HFrEF, in postmenopausal women [29].

One should not forget the importance of coronary microvascular dysfunction and ischemia on HFpEF [38]. The ARIC study (the Atherosclerosis Risk in Communities Study) recently showed that retinal microvascular dysfunction was a long-term risk factor of mortality and ischemic stroke in both sexes, while microvascular heart disease was an independent predictor only in women, but not in men [39]. 

#### 3.3.5. Atrial Fibrillation

In atrial fibrillation, women seem to have a larger LA volume index and worse LA pump function than men [40]. Furthermore, atrial fibrillation seems to increase the risk of HF mostly in women, but not in men [41]. The TOPCAT trial (Treatment of Preserved Cardiac Function HF with an Aldosterone Antagonist Trial) revealed that atrial fibrillation was related to the risk of hospitalization in both sexes with HFpEF, but more strongly in women [42]. Lam et al. showed the association between the sexes regarding atrial fibrillation, lower cardiorespiratory fitness, natriuretic peptide elevation, and left atrial enlargement in HFpEF [43]. The authors concluded that atrial fibrillation in women might be associated with a greater risk of adverse events than in men. This is yet to be clarified, because other investigations have denied the role of atrial fibrillation in HFpEF in women and reported similar prevalence of this arrhythmia between the sexes [36], or even a higher prevalence in men [6,7]. 

### 3.4. Race

The influence of race on the development and outcome of HFpEF in the sexes has not been investigated in detail so far, but initial observations point to a higher risk of HFpEF in white women. Eaton et al. conducted a comprehensive analysis of influence of race on HFpEF and HFrEF development, as well as the role of various risk factors in each race [29]. The analysis was performed in a large multiracial cohort of 42,170 postmenopausal women. The findings revealed that white race, and not African American and Hispanic groups, was associated with both, HFpEF and HFrEF [29]. Obesity was reported as a more important risk factor of HFpEF in African American women in comparison with white women. There was some difference in the prevalence of potentially modifiable risk factors among women with HFpEF [29]. In white women, about 66% of the population-attributable risk percentage was related to hypertension and obesity, while diabetes and coronary artery disease made up 25% of this risk [29]. For African American women, hypertension and obesity were associated with >90% of the population-attributable risk percentage, whereas for Hispanic women, the same risk factors were associated with approximately 72% of the population-attributable risk percentage [29].

A large cohort study which followed 12,417 participants for almost 12 years showed that the lifetime risk of overall HF was higher in non-blacks than blacks [10]. The lifetime risk of HFpEF development was approximately 1.5-fold higher in non-blacks as compared with blacks. Blacks had a similar lifetime risk of HFpEF and HFrEF, whereas non-blacks had a higher risk of HFpEF than HFrEF [10]. However, the authors did not present sex-specific analyses.

### 3.5. Other Risk Factors

Goyal et al. found that chronic renal and hepatic failure was more prevalent among men with HFpEF [6]. Harada et al. confirmed these findings in HFpEF patients [7]. Another recent study reported that anemia was an independent predictor of all-cause mortality in women with HFpEF, but not in those with HFrEF [44]. On the other hand, Duca et al. did not find any difference whatsoever in the prevalence of chronic kidney disease between women and men with HFpEF [8].

Anemia was described as an additional risk factor of HFpEF development in women, but not in men [6]. Eaton et al.—in a large study that included only postmenopausal women—reported that anemia was an independent predictor of HFpEF occurrence in African American women, but not in white women [29]. There have also been studies that showed a borderline higher prevalence of anemia among men with HFpEF [8].

Chronic pulmonary obstructive disease represents a risk factor of HFpEF in both sexes. Goyal et al. showed that it is more prevalent in men with HFpEF older than 75 years of age, and in women with HFpEF younger than 75 years of age [6]. Eaton et al. found that chronic lung disease was an independent predictor of HFpEF only in white, but not in African American and Hispanic women [29]. Duca et al. reported higher prevalence of chronic obstructive pulmonary disease in men with HFpEF [8].

In a population of older patients who underwent radiation therapy due to breast cancer, cardiac radiation exposure increased the risk of HFpEF development, and the higher mean cardiac radiation dose was associated with a greater risk of HFpEF [45]. The radiation was associated more strongly with HFpEF than with HFrEF occurrence. 

In summary, the pathophysiological studies available so far consistently show that obesity, hypertension, and diabetes have sex-specific effects in HFpEF, whereas for other risk factors, there is some evidence, but it is not so clear.

## 4. Clinical Studies

In the last few years, important studies regarding hemodynamic changes and LV remodeling in HFpEF have been published [7,37,46,47]. However, only a small portion have been devoted to sex-related changes [7,47]. Our study group has recently published data regarding the diagnosis of HFpEF using diastolic stress echocardiography testing, which also partly illuminates the hemodynamic changes typical of HFpEF [48].

### 4.1. Hemodynamic Changes and Cardiovascular Remodeling

A recently published study found that women with HFpEF had worse diastolic reserve, estimated by: (i) elevated LV filling pressures measured by echocardiographic and invasive measurements at exercise; (ii) lower systemic and pulmonary arterial compliance; and (iii) worse peripheral oxygen kinetics [47]. This elegant investigation included 161 HFpEF subjects (114 females and 47 males) who had already been diagnosed and treated for HFpEF. The authors performed right heart catheterization and revealed that females had a higher pulmonary capillary wedge pressure indexed to peak exercise workload, and lower systemic and pulmonary arterial compliance at exercise [47]. Interestingly, the significant difference in right atrial pressure, pulmonary artery pressure, wedge pressure, stroke volume, and stroke volume index between the sexes was not present at rest [47]. Only through exercise was it possible to reveal increased LV filling pressures in women, but not in men with HFpEF. Echocardiographic findings demonstrated differences between the sexes at rest. Women had higher LV filling pressure, evaluated by E/e’ ratios at rest, and more pronounced at peak exercise, together with a higher LVEF and smaller ventricular dimensions [47]. On the other hand, mitral E/A ratio was similar between the sexes at rest and during exercise. Women showed significantly higher systemic and pulmonary vascular resistance levels (indexed to BSA (Body surface area)) both at rest and during exercise [47]. Correspondingly, both systemic and pulmonary compliance levels were lower in women at rest and during exercise. Arterial elastance was significantly higher in women during exercise in comparison with men, although statistical significance vanished after indexing to BSA [47]. Ventricular–vascular coupling was reduced in women compared to in men after indexing to BSA at rest and after exercise. However, the difference in ventricular–vascular coupling did not reach statistical significance after exercise. The authors did not report significant sex differences between baseline or exercise in mixed venous oxygen saturation, arteriovenous oxygen differences, oxygen consumption levels, or oxygen exchange ratios [47].

This study agrees with our findings that showed usefulness of diastolic stress echocardiography in discovering HFpEF patients in the individuals with dyspnea on exertion in simple clinical settings [48], without the use of right heart catheterization and comprehensive blood gas analysis.

Duca et al. did not find any difference in invasive hemodynamic parameters between women and men with HFpEF, except borderline higher wedge pressure in women and significantly increased diastolic pressure gradient in men [8].

The PARAMOUNT trial (Prospective comparison of ARNi with ARB on management of heart failure with preserved ejection fraction) was the first investigation that provided a detailed sex-specific analysis of LV structure, function, and mechanics in HFpEF [37]. As expected, LV mass and volumes indexed for body size were significantly lower in women with HFpEF. However, left atrial volumes indexed for height and LVEF were significantly higher in women [37]. There was no difference in mitral E/A ratio, but E/e’ was significantly higher in women [37]. Interestingly, there was no difference in LV longitudinal, circumferential, and radial strain between the women and men with HFpEF [37]. The authors showed a trend towards abnormal LV geometry (concentric remodeling, eccentric and concentric LV hypertrophy) in women, but not in men. The authors reported that effective arterial elastance, LV end systolic elastance, and diastolic stiffness were higher among women with HFpEF, whereas ventricular–vascular coupling was similar between the sexes [37].

Harada et al. reported similar findings regarding smaller LV and better LVEF in women with HFpEF, but E/e’ ratio was similar between the sexes and the left atrium was larger in men [7]. Concentric LV hypertrophy was predominant in women, whereas eccentric LV hypertrophy was more prevalent in men with HFpEF [7]. The findings from hemodynamic and echocardiographic studies are summarized in Table 2.

### 4.2. Outcomes

Goyal et al. demonstrated that women with HFpEF had lower in-hospital mortality compared with men (4.2% vs. 4.6%, *p* < 0.001) [6]. Interestingly, the sex difference in mortality was only seen in women older than 75 years of age, whereas women younger than 75 years of age had similar mortality as men. The TOPCAT trial showed no difference in outcomes (cardiovascular and all-cause mortality), or in hospitalization due to heart failure, between women and men with HFpEF [9].

Nakada et al. revealed that the incidence of cardiovascular death and admission due to HF tend was lower in female than in male HFpEF patients, but the difference was not statistically significant [36]. A small cohort study that involved 260 HFpEF patients and followed them for 30 months showed that men had higher rates of cardiac death (16.5% vs. 6.1%, *p* = 0.008) and lower rates of non-cardiac death (2.5% vs. 10.5%, *p* = 0.030) in comparison with women [8]. Male sex was independently associated with cardiac death. An investigation that included 4161 HFpEF patients (67% women) hospitalized due to HF symptoms demonstrated no difference between women and men in in-hospital mortality, as well as in 30 day and 180 day outcomes [49]. Hsich et al. involved 37,699 patients with HFpEF (65% women and 35% men), and did not find any sex difference in in-hospital mortality (2.61% in women vs. 2.62% in men, *p* = 0.96) [50].

## 5. Treatment

There is no current consensus on the optimal therapy for HFpEF, and it is unknown if it should be treated in the same way as HFrEF or as a completely different entity. The data on this topic are scarce, and there is even less knowledge of the benefit of sex-specific therapy in HFpEF. Our study group recently summarized the sex-specific therapeutic approach to arterial hypertension [51]. Considering the fact that hypertension represents almost “sine qua non” in the HFpEF continuum, this approach could serve as a good starting point for sex-specific treatment of HFpEF. Table 3 shows known pathophysiological mechanisms responsible for higher prevalence of HFpEF among women (hormonal, bio-hormonal, risk factors, and race), as well as potential targets for therapy in these patients.

The TOPCAT trial showed that spironolactone had a similar effect on the primary outcome in HFpEF in both sexes [9]. However, spironolactone-associated reduction in all-cause mortality was observed only in women, with a significant interaction between the sex and the treatment arm [9].

Several studies did not report differences in the prevalence of different medications between women and men with HFpEF [7,8], even though Harada et al. showed a higher prevalence of beta-blocker use in men with HFpEF [7]. However, these investigations were not designed to investigate the influence of different medications on the outcome in HFpEF.

A large meta-analysis that included 28,636 HFpEF patients (35–70% women) showed a significant benefit from the use of beta-blockers on all-cause mortality in observational studies, with a reduction in mortality by 21% in HFpEF patients, irrespective of sex [52]. However, this was not confirmed in randomized trials [52]. 

Khan et al. used the data of randomized trials involving 17,284 HFpEF patients to investigate the effect of ACE inhibitors and angiotensin receptor blockers on outcome [53]. In this pooled analysis, RAAS inhibitors did not show any effect on all-cause mortality, while the results from observational studies showed a significant improvement. In pooled analyses of all studies, angiotensin-converting enzyme inhibitors showed a reduction of all-cause mortality [53]. There was no reduction in cardiovascular mortality; however, in the pooled analysis of randomized trials, there was a trend towards reduced HF hospitalization risk, but it did not reach any statistical significance [53].

Spironolactone has been shown to improve functional capacity and LV diastolic function in a study of HFpEF patients [54]. However, a sex-specific subanalysis was not performed in this study. Another small prospective observational study showed no correlation between soluble neprilysin and outcome in patients with HFpEF [55]. The PARAGON-HF trial (Prospective Comparison of Angiotensin Receptor Neprilysin Inhibitor with Angiotensin Receptor Blocker Global Outcomes in HFpEF) was designed to compare the effects between sacubitril/valsartan and valsartan in reducing morbidity and mortality in HFpEF [56].

## 6. Conclusions

HFpEF represents the most prevalent form of HF in women, which is associated with adverse clinical outcomes. HFpEF has unique pathophysiology in women related to certain comorbidities and specific cardiovascular remodeling. Some risk factors such as arterial hypertension, obesity, diabetes mellitus, coronary artery disease, atrial fibrillation, and race have specific features that might be responsible for a particular phenotypic profile in women with HFpEF. These risk factors are associated with specific cardiovascular changes—left ventricular diastolic dysfunction and hypertrophy, ventricular–vascular coupling, and impaired functional capacity—that are related to particular cardiac phenotype and HFpEF development. Sex hormones are still considered to have the leading role in sex-specific cardiovascular remodeling, including HFpEF. There is no agreement regarding outcome in women with HFpEF. Sex-specific HFpEF treatment is far from a reality at this moment, but it represents a very important future direction toward personalized medicine. A majority of the studies that have investigated the outcome or treatment in HFpEF patients did not aim to research the differences between the sexes. Therefore, longitudinal studies with sex-specific outcomes as the primary aim should be conducted.

## Figures and Tables

**Table 1:** Sex-specific differences in risk factors in HFpEF patients.

Reference	Sample Size	Women/Men (%)	Study Type	Main Findings
Goyal et al. [6]	1,889,608 pts hospitalized for HFpEF	1,208,763 (64)	Short follow-up	Arterial hypertension, obesity, and anemia were significantly more prevalent among women than men with HFpEF. Diabetes was more prevalent in women younger than 75 years and in men older than 75 years. Atrial fibrillation and coronary artery disease were more prevalent in men.
Harada et al. [7]	733 HFpEF pts	529 (72)	Cross-sectional	Obesity (BMI > 25 kg/m^2^), diabetes, coronary artery disease and atrial fibrillatio were more frequent in men than in women with HfpEF.
Duca et al. [8]	260 HFpEF pts	181 (70)	30 month follow-up	No difference in cardiovascular risk factors between women and men with HFpEF, except smoking and chronic obstructive lung disease.
Pandey et al. [10]	12,417 subjects	6854 (55.2)	11.6 year follow-up	The lifetime risk of HFpEF did not differ between women and men.
Eaton et al. [29]	42,170 postmenopausal women	All	13.2 year follow-up	Hypertension, diabetes, and obesity were independent predictors only of HFpEF, but not HFrEF. The white race, and not African American and Hispanic, was associated with both, HFpEF and HFrEF.

BMI—body mass index, HFpEF—heart failure with preserved ejection fraction, HFrEF—heart failure with reduced ejection fraction.

**Table 2:** Sex-specific differences in hemodynamic changes and cardiovascular remodeling in HFpEF patients.

Reference	Sample Size	Women/Men (%)	Study Type	Main Findings
Beale et al. [47]	161 HFpEF pts	114 (71)	Cross-sectional	Women with HFpEF had worse diastolic reserve. LV filling pressures measured by echocardiographic and invasive measurements at exercise were higher than in men. Women showed lower systemic and pulmonary arterial compliance, as well as worse peripheral oxygen kinetics.
Harada et al. [7]	733 HFpEF pts	529 (72)	Cross-sectional	Females with HFpEF had smaller LV diameters and better LVEF. LV filling pressure was similar between sexes. Left atrium was larger in men. Concentric LV hypertrophy was predominant in women, and eccentric in men with HFpEF.
Duca et al. [8]	260 HFpEF pts	181 (70)	30 month follow-up	No difference in invasive hemodynamic parameters between women and men with HFpEF. LV mass index was significantly higher in men, and LVEF measured by CMR was significantly higher in women.
Gori et al. [37]	279 HFpEF pts	159 (57)	3 year follow-up	Indexed LV mass and volumes were significantly lower in women with HFpEF. Indexed left atrial volume, LVEF and LV filling pressure were significantly higher in men. There was no difference in LV longitudinal, circumferential, and radial strain between women and men with HFpEF. Effective arterial elastance, LV end systolic elastance and diastolic stiffness were higher among women with HFpEF.

HFpEF—heart failure with preserved ejection fraction, LV—left ventricle.

**Table 3:** Summarized pathophysiology and therapy in women with HFpEF.

Hormonal	Bio-Hormonal	Risk Factors	Race	Therapy
Decreased estradiol	Higher angiotensin-converting enzyme serum activity in women	Obesity	White race, and not African American and Hispanic, was associated with HFpEF	Spironolactone-associated reduction in all-cause mortality was observed only in women
		Hypertension		
Higher testosterone	Increased sympathetic nervous system activity in women	Diabetes	Obesity was reported as more important risk factor in African American women	Sex-specific differences regarding beta blockers and renin-angiotensin inhibitors in HFpEF have not been investigated so far
		Coronary heart disease		
	Decreased nitric oxide bioavailability	Atrial fibrillation		
		Anemia		
	Increased prostaglandin and prostacyclin levels	Chronic obstructive pulmonary disease		
	Oxidative stress	Renal dysfunction		
		Chemo- and radiotherapy		

HFpEF—heart failure with preserved ejection fraction.
